# From second thoughts on the germ theory to a full-blown host theory

**DOI:** 10.1073/pnas.2301186120

**Published:** 2023-06-12

**Authors:** Jean-Laurent Casanova

**Affiliations:** ^a^HHMI, New York, NY 10065; ^b^St. Giles Laboratory of Human Genetics of Infectious Diseases, The Rockefeller University, New York, NY 10065

**Keywords:** René Dubos, germ theory, host genetics, inborn errors of immunity, infectious diseases

## Abstract

In 1955, René Dubos famously expressed his “second thoughts on the germ theory”, attributing infectious diseases to various “changing circumstances” that weaken the host by unknown mechanisms. He rightly stressed that only a small minority of individuals infected by almost any microbe develop clinical disease. Intriguingly, though, he did not mention the abundant and elegant findings reported from 1905 onward that unambiguously pointed to host genetic determinants of infection outcome in plants and animals, including human inborn errors of immunity. Diverse findings over the next 50 y corroborated and extended these earlier genetic and immunological observations that René Dubos had neglected. Meanwhile, the sequential advent of immunosuppression- and HIV–driven immunodeficiencies unexpectedly provided a mechanistic basis for his own views. Collectively, these two lines of evidence support a host theory of infectious diseases, with inherited and acquired immunodeficiencies as the key determinants of severe infection outcome, relegating the germ to an environmental trigger that reveals an underlying and preexisting cause of disease and death.

## Foreword

### René Dubos.

Let’s start with a disclaimer: René Dubos (1901 to 1982) is one of my heroes. Not only was he a bold, multitalented scientific explorer, a legend in the fields of microbiology and biology, but also a broad and deep thinker, a giant in the fields of ecology and humanities (1). Among his many achievements, he was the first to search for and discover antibiotics (2), he discovered the intestinal microbiome (3), and he wrote one of the most profound intellectual biographies of Louis Pasteur (4) as well as a fascinating history of tuberculosis (5). I admire him greatly, and very few scientists or philosophers have had a stronger influence on me. It is not my intention here to attack him unfairly. That would also be treacherous on three counts, as, almost a century later, I, like him, am a Frenchman in New York, a faculty member at Rockefeller University, and on the editorial board of the *Journal of Experimental Medicine*. I hope that he would see my criticisms as fair. I have even borrowed here the brilliant title of his paper published in 1955 in the *Scientific American*: “Second thoughts on the germ theory” (6). I have done this not only because this is one of the catchiest titles I have come across in my career but also because I intend to discuss why his second thoughts on the germ theory are of historical and philosophical importance. In my eyes, Dubos’ second thoughts are important not only through what the words he chose to use express so eloquently but also because of the surprising implications of what he did not say. Like Dubos, I am not a professional historian or philosopher of science. As such, I undoubtedly display some of the shortcomings of any amateur. However, I have at least tried to avoid the pitfalls of anachronism and presentism (7, 8). Moreover, my own scientific endeavors provide me with a perspective on Dubos and infectious diseases that differs in many ways from those of historians and philosophers.

### Dubos’ Views.

Dubos lucidly drew the attention of his readers to the puzzling observation that only a small minority of infected people die from infection or even develop clinical disease. Silent infection, including Charles Nicolle’s “inapparent infections” (replicating microbes in asymptomatic hosts) (9) and Clemens von Pirquet’s “latent infections” (dormant microbes in asymptomatic hosts) (10), is the rule, and it applies over the whole spectrum of living species. For most infections, disease is the exception to the rule. This situation is referred to here as the “infection enigma”. Even in the absence of medical care, no more than a dozen microbes are currently capable of killing more than 10% of the people they infect (11–15) (*SI Appendix*, Fig. S1). In line with his ecological vision of the world, Dubos suggested that diverse “changing circumstances” ranging from an “unhappy love affair” to “life in a concentration camp” might weaken the host, serving as the key determinants of disease and death from infection. Dubos considered these changing circumstances to be capable of accounting for the immense interindividual and intraindividual clinical variability during infection. However, he acknowledged that the underlying mechanisms were unknown and argued for their study and discovery. Provocatively, he claimed that immunological knowledge, limited at the time to antibodies and their set of accompanying serum proteins forming the “complement”, is irrelevant to this phenomenon. More surprisingly, he did not write a single word on the 50-y-long streak of studies on the host genetics of infectious diseases, including not only pioneering studies published decades previously by some of his colleagues at Rockefeller but also human genetic studies that clearly attributed infectious diseases to certain inherited molecular or cellular immunological deficits. In this essay, I analyze Dubos’ 1955 paper in the light of host genetic studies of immunity to infection conducted since 1905. I then review the development of the field of inherited immunodeficiencies, and that of acquired immunodeficiencies, in the 50 y or so following the publication of Dubos’ paper. Finally, I speculate as to why a great mind like René Dubos asked the right question, but then focused on hypothetical acquired deficits of host defense, the mechanistic basis of which was unknown at the time, ignoring solid factual data for inherited deficits, which had already been documented across multiple species, including humans, and, in some cases, characterized immunologically. These findings would have given much more weight to his host theory of infectious diseases. Perhaps this is precisely the step that Dubos was not ready to take.

### Second Thoughts on the Germ Theory

#### Dubos and Pasteur.

René Dubos wrote his second thoughts on the germ theory in 1955, about 60 y after Pasteur’s death in 1895 and 70 y after the general acceptance of the germ theory around 1885 (16, 17). Dubos had already written a brilliant and insightful intellectual biography of Pasteur, “Pasteur, a freelance of science”, which was published in 1950 (4). This book may even surpass “The history of a mind”, which I and many others see as a deeply profound, intimate, first-hand description of Pasteur’s mind at work, written by his chief disciple, Emile Duclaux (18). Dubos’ analysis of Pasteur is interesting in many ways, particularly because of his poetic reflections on what Pasteur might have done, had he made different choices at the many crossroads in his extraordinary scientific career. This notion is relevant to this essay, in which I ask why Dubos did not consider findings and ideas pertaining to host genetics of infectious diseases that were widely available when he wrote his 1955 paper. He completely ignored this line of research, just as he had in 1950, when he could have speculated about the possible course of the germ theory had Pasteur opted to pioneer host studies, after he reported in 1870 that one of the two infectious conditions of silkworm he was studying, flacherie, is “transmitted from the parents to the offspring, not in the sense that the microbe is transmitted, but in the sense that predisposition to disease is transmitted” (19). Pasteur’s studies of two infections of silkworm, pébrine and flacherie, established the germ theory. Had Pasteur read the work of Mendel, who published his breakthrough discoveries of the laws of genetics in 1865, right at the start of Pasteur’s research on silkworms (20), maybe he would have pursued this line of research by focusing his subsequent efforts on the host component of flacherie. Dubos did not consider this alternate history in his essay on Pasteur and made no mention of host genetics in his 1955 paper.

#### The Infection Enigma.

So what did Dubos say in his famous *Scientific American* paper (6)? Its title is explicit and could hardly be more provocative, as the germ theory was at the time, and has remained, by far the greatest medical theory, a theory that has explained why life expectancy at birth remained at about 20 to 25 y, worldwide, from the dawn of humankind until the advent of hygiene, vaccines, aseptic surgery, and antibiotics, with half of all children dying from unexplained fevers before the age of 15 y (14, 21). This theory also provided active means to prevent and cure fevers. Prevention was achieved by Pasteur himself, in 1881, with the triumph of vaccination (19), and cure was achieved in 1932 with the synthesis of the first anti-infectious agents, sulfamides, by Gerhard Domagk (22). The germ theory also solved a problem posed across all animal and plant species. The short abstract of the paper neatly complements its title: “Everyone harbors disease germs, yet not everyone is sick. This suggests that germs are less important in disease than other factors affecting the condition of the host”. Dubos then opens his essay by posing as a firm supporter of the germ theory, regretting that there are still opponents to the germ theory, and perhaps magnifying their social importance to distinguish their blind skepticism or hostility more clearly from his own reasoned doubts and nuances. Despite this rhetorical precaution, openly divulging his “second thoughts” in 1955 probably sounded, in serious scientific circles, especially among his own community of microbiologists, like a physicist raising doubts about Newton’s law of gravity. Overall, the title and abstract pose what is certainly the key (but neglected) problem in the field of infectious diseases—the infection enigma—and they do so in an unprecedentedly provocative manner. However, in the introduction, Dubos prudently attempts to convince the reader that he should not be mistaken for a heretic.

### Changing Circumstances.

René Dubos rightly goes on to point out that most infections are silent in most people, illustrating this observation with three examples: herpes simplex virus and cold sores, staphylococcus and skin abscesses, and the mycobacterial agent of tuberculosis. He insisted that these and many other infections remain silent for long periods in the individuals eventually diagnosed with them. He then logically argued that as these infectious diseases occur only at a certain, distant time in someone’s infectious history, they must attest to a recent causal “weakening” of the sick host, after infection, but before the development of disease. He further argued, precisely because the patient was already infected but had remained healthy, that this weakness is not only recent but also results from changing circumstances. By analyzing the various risk factors documented observationally or experimentally in humans and animals, respectively, he evoked circumstances as diverse as irradiation, malnutrition, diabetes, life in prison, overwork, a sentimental drawback, menstruation, surgery, antibiotics, poor diet, or any other “stress” as probable causes. Central to his theme is the notion that the host with an infectious disease is, paradoxically, “constitutively resistant”. For Dubos, the patient is a previously resistant individual weakened only recently by stress, due to a change of circumstances, mostly environmental in nature. He, thus, considered these changes to be “ecological” in nature because, although they affect their host, they originate from the environment, and may include modifications to the environment by the host, through the administration of antibiotics that modify the gut microbiome (an internal environment), for example. His changing circumstances are modifications of the ecosystem, whatever their nature, with an impact on the host.

### Elusive Mechanisms.

Dubos noted both the impact of antibiotics on the intestinal flora and that of irradiation on the intestinal barrier. Nevertheless, he acknowledged that the mechanisms weakening host defense were largely unknown. Consistent with the period, during which immunology was primarily an “immunochemistry” (23), Dubos ruled out antibodies and their complement as contributing factors because of the documented presence of pathogen-specific antibodies in both healthy and sick individuals. Instead, he took the examples of Alexander Fleming’s lysozyme, discovered in 1928 (24), and Louis Pillemer’s properdin, discovered in 1954 (25), as possibly contributing to host defense and being affected by certain risk factors. He saw these molecules as “outside” the realm of “immunology”, a common view at the time, even for properdin, the action of which was thought to be dependent on complement, but, paradoxically, independent of antibodies. This remained the case long after the birth of “immunobiology” in the 1960s with the discovery of antibody-producing and bone marrow– or bursa of Fabricius-derived B lymphocytes and thymus-derived T lymphocytes. Both topics of investigation were dealt with immunologically much later, when, after a tragic controversy, properdin eventually came to define the alternative pathway of complement activation (26), and when the study of innate immunity, including lysozyme, at last gained traction among immunologists (27). René Dubos speculated that these two candidate molecules, or others, might be transiently downregulated, by irradiation or other circumstances. The lack of a description of the mechanisms underlying infectious diseases arguably did not help Dubos defend his theory of changing circumstances, despite solid observational and experimental evidence. Perhaps more problematically, Dubos proposed an ecological hypothesis, based on the observation of longitudinal intraindividual clinical variability (people becoming sick after a long period of silent infection), to account for the immense interindividual clinical variability in the course of infection (a minority of infected people developing disease). This idea suffers from the inherent weakness that, in any given population of individuals enduring the same environmental modifications, interindividual clinical variability persists.

### An Ecological Theory.

Before discussing what Dubos omitted from his ecological theory, namely a host genetic theory of infectious diseases, let us consider the even more intriguing absence of any microbial theory in his paper. Surprisingly for an eminent microbiologist, Dubos did not consider variations of microbial virulence or inoculum as potential drivers of infectious diseases, despite the already ample documentation of the effects of these two factors in animal models, albeit admittedly without observational confirmation in humans (28). Maybe he thought that natural inocula did not differ much between individuals or that the inoculum was too far back in the patient’s history to be relevant? For herpes viruses or *Mycobacterium tuberculosis*, for example, the initial inoculum probably has no effect on disease occurring after 5 or 10 y of silent infection. Dubos probably thought that differences in virulence might account for interpopulation but not interindividual clinical heterogeneity. Or maybe he was, after all, genuinely trying to find host explanations for infectious diseases outside of the theme of the germ theory and its many qualitative (virulence) or quantitative (inoculum) variations. René Dubos boldly posed the key scientific problem in the field of infectious diseases, a problem first posed by others, especially Charles Nicolle, who famously asserted in his 1928 Nobel speech that “This new concept of inapparent infections that I introduced to pathology is, without a doubt, the most important of the discoveries that I was able to make”. Nearly 30 y later, Dubos proposed an ecological explanation of the infection enigma. His proposal was based on many observational and experimental observations, in humans and animals, but suffered from two major failings. First, his theory of interindividual variability was based solely on observations of intraindividual variability. Second, it fell short of providing any molecular, cellular, or tissue-based mechanisms, which would have bolstered plausibility, not to mention causality, when proposing an alternative theory, or even a theory complementary to the germ theory, the establishment of which was a painful process, with progress via many epic battles.

## A Few Thoughts about Host Genetics and Immunity

### Dubos’ Omissions.

I think that Dubos’ 1955 paper is even more interesting in terms of the ideas and facts that he did not mention or even allude to. Indeed, in this three-column five-page dissertation, not a single mention is made of host genetics and host immunity. Immunology is executed in a single sentence: “The classical doctrines of immunity show no light on precisely what mechanisms determine whether dormant microbes will remain inactive or begin to act up”. The words “genetics”, “heredity”, “inherited”, and “hereditary” are not even mentioned. The absence of host genetics in Dubos’ paper is particularly striking because the Mendelian basis of fungal infection in wheat was unambiguously documented as early as 1905 (29). This paved the way for Harold H. Flor and other plant geneticists to propose and document the revolutionary gene-for-gene concept, with each plant resistance gene corresponding to a microbial virulence gene (30). By 1955, this theory had already been unequivocally proved, by means of classic genetics, and had been amply reviewed. In defense of Dubos, maybe he did not consider plants because of their considerable evolutionary distance from humans. However, other studies from the late 1920s onward unambiguously demonstrated the key role of genetic background in the outcome of infection in animal species as diverse as mice, guinea pigs, and rabbits (11) (*SI Appendix*, Fig. S1). Dubos did not cite any of these compelling studies, including the seminal mouse studies published by his colleague at Rockefeller University, Leslie T. Webster, in the *Journal of Experimental Medicine*, of which he was a prominent editor (31–37), and rabbit studies conducted by Max B. Lurie on Dubos’ preferred infection for study, tuberculosis (38). Dubos had brilliantly reviewed the history and challenges of tuberculosis in “The white plague”, which he published in 1952 (5). That same year, Lurie extended his observations on the genetic basis of rabbit tuberculosis in the *Journal of Experimental Medicine* (39).

### Human Genetics.

Again, it could be argued that Dubos intended to restrict his discussion to humans. However, as far back as 1909, the “biometrician” Karl Pearson had already reported genetic epidemiological observations and equations suggesting that tuberculosis has a strong human genetic basis (40). At the other end of the genetic spectrum, “Mendelian” geneticist Archibald Garrod devoted an entire chapter of his 1931 landmark treatise on “Inborn factors in disease” to the human genetic basis of infectious diseases (41). Garrod was also familiar with immunological concepts and asserted that “It is, of necessity, no easy matter to distinguish between immunity which is inborn and that which has been acquired”. Moreover, there were many reports of familial clustering and seemingly hereditary predispositions to infection. These atypical, if not heretical studies of infectious diseases, amusingly often published in *The Journal of Heredity*, were admittedly not conclusive because contagion may provide an alternative explanation for the clustering of infectious diseases. One notable exception is bacterial appendicitis, which is infectious but not contagious. There have been many reports of familial clusters of appendicitis suggestive of a hereditary predisposition, but the publication by Sister Flavia deserves a special mention for its detailed description and visionary discussion (42). A critical step forward was made with twin studies, which compared the concordance rate of specific infectious diseases in monozygotic (identical) and dizygotic (fraternal) twins. In particular, twin studies of tuberculosis showed, both in Germany in the 1930s (43) and the United States in the 1940s (44), that tuberculosis status was much more concordant in monozygotic (90%) than in dizygotic twins (20%). René Dubos did not cite any of these compelling studies, including the classic 1943 paper by Kallman and Reisner reporting a study conducted on the East Coast of the United States.

### Inborn Errors of Immunity (IEI).

It could be surmised that a lack of molecular or cellular data prevented Dubos from giving these epidemiological or clinical genetic studies the credit they deserved. However, there were already enough studies of human IEI to argue against this view. First, the epidermodysplasia verruciformis described by Wilhelm Lutz was already known, by 1946, to be an autosomal recessive predisposition to flat wart-causing viruses (45). The causal viruses were, however, not discovered until the 1970s, and the patients displayed no hematological or immunological abnormalities (*SI Appendix*, Fig. S1). By contrast, alymphocytosis was first described in 1950 (46), but the role of lymphocytes in host defense, including that mediated by the control of antibody production, was not discovered until the 1960s. In this respect, Rolf Kostmann’s description of autosomal recessive neutropenia in 1950 (47) and Ogden Bruton’s description of X-linked recessive agammaglobulinemia in 1952 (*SI Appendix*, Fig. S1) are more compelling. These two studies propelled the field of human genetics of infectious diseases into the molecular and cellular era. They unequivocally attributed severe staphylococcal and pneumococcal disease to a genetically determined lack of blood neutrophils and gammaglobulins, respectively. Admittedly, Kostmann’s first paper was published in Swedish in 1950, and an English version did not appear until 1956 (47, 48). However, Bruton’s seminal publication was rapidly followed by independent reports, particularly by David Gitlin who, like Bruton, was working on the East Coast of the United States (49–51). Studies published before 1955, thus, clearly provided molecular and cellular evidence that immunity to infection in humans can be genetically controlled. They documented that Mendelian IEI can underlie life-threatening infectious diseases and, therefore, that human infectious disease can be due to human genetic lesions impairing a specific arm of immune responses. It is remarkable that Dubos does not cite any of these studies.

### The Sickle Cell Trait.

We can speculate that he might have seen patients with Mendelian IEI simply as rare outliers, the observation of which would not reveal any valid notion pertaining to infections in the general population. This is possible, as the neglect of rare diseases has always been prominent in certain circles with a conscious or unconscious typological imprint, resulting in a reluctance to approach biological and medical problems from a nominalist angle. The fundamentally nominalist views of living organisms espoused by the founders of the two branches of biology, evolution and physiology, Charles Darwin and Claude Bernard, are, paradoxically, often ignored, neglected, or misunderstood, even among modern biologists who have perhaps been excessively influenced by physicists. Typologists tend to think of nature as a collection of inert items, characterized by types and governed by laws, whose fate is therefore predictable, while nominalists tend to think of the living world as an infinite diversity of unique organisms, whose evolution by natural selection is unpredictable. This bias would, however, be surprising for the microbiologist–ecologist René Dubos, whose scientific work was clearly more inspired by the diversity of living organisms rather than their unity (1). Whatever Dubos’ reasons for not citing papers reporting rare patients, he also failed to cite papers reporting studies on large populations. Indeed, population-based studies were undertaken in parallel with patient-based studies. In 1954, Anthony C. Allison reported the landmark discovery that the sickle cell trait provides large African populations with 10-fold greater protection against the risk of cerebral malaria, attesting to natural selection by pathogens at the molecular level and at a gigantic scale, while also documenting a genetic and mechanistic resistance to a common infection in countless individuals (*SI Appendix*, Fig. S1). Allison’s breakthrough is nowhere to be found in Dubos’ paper, no more than John B.S. Haldane’s previous suggestion that malaria may select alleles that are expressed in human erythrocytes and render their carriers resistant to it (52). Dubos wrote in his essay that “natural resistance stems in part from evolutionary selection of the hosts best endowed with mechanisms for withstanding the infections”. He saw evolution as a testimony in the present of what happened in the past, an explanation of universal resistance, rather than as an ongoing and perpetual driver of interpopulation or interindividual variability during infection. Thus, human genetic findings at the molecular and cellular levels, both in individual patients and large populations, were available to Dubos, but he chose not to discuss or even mention any of them.

### A Lifetime View.

Evidence that host genetics controls infection outcomes across plants and animals, including humans, had, thus, been published regularly in diverse journals from 1905 onward. Nevertheless, René Dubos did not mention any of these discoveries published over the course of half a century. He did not cite any of the many impactful studies supporting a congenital host theory of infectious diseases. Before reviewing progress in the field over the 50 y that followed, and before judging Dubos’ 1955 paper in the light of what was known in 1955 and 2023, we should pause for a second and exclude two obvious reasons why Dubos would not have cited these studies. First, we cannot seriously envisage the possibility that Dubos did not have access to these publications. Many of these studies were reported by colleagues of his on the East Coast of the United States, even at the Rockefeller University itself, and more than once in *The Journal of Experimental Medicine*. Others dealt with tuberculosis, a disease that Dubos studied for half a century. He invented the famous “Dubos medium” for growing *Mycobacterium tuberculosis* in vitro. A book entitled “Genetic predisposition to tuberculosis” had even been published at Harvard University Press in 1944 (53). We know that Dubos had read this book, as he quoted it in his own book about tuberculosis, briefly discussing the potential contribution of human genetics (5). It also seems very unlikely that the 1955 *Scientific American* paper was an isolated moment in Dubos’ bibliography, perhaps motivated by the broad nature of the readership of this journal. On five other occasions, in publications appearing between 1954 and 1975 and reflecting on the pathogenesis of infectious diseases, Dubos did not mention genetics even once (54–58). The recurrent theme in all these papers remains the same: changing circumstances of an ecological nature weaken the host and account for the sudden onset of disease in a previously healthy, constitutively “resistant” carrier of the pathogen.

## New Thoughts about Inherited and Acquired Immunodeficiencies

### Immunosuppression.

However, Dubos was nevertheless prescient, because his main hypothesis of changing circumstances gained considerable support in the following 50 y. It did not do so through a clarification of the mechanisms by which any of the circumstances he had listed operated. Instead, it gained support via two unpredictable changes of circumstances, the impact of which was quickly and thoroughly deciphered mechanistically. Immunosuppression for organ transplantation and chemotherapy for the treatment of cancers became widely available in the 1960s (*SI Appendix*, Fig. S1). In some ways, they can be seen as an extension of cortisone therapy, which had begun in the early 1950s (59) and, with time, was found to favor certain infectious diseases, as subsequently acknowledged by Dubos (60). These new treatments saved lives, but at the price of creating a predisposition to life-threatening infections. They showed that decreases in the counts and/or function of lymphoid and/or myeloid blood leukocytes predisposed patients to severe infections, including infections seen only very rarely or not all in the general population. Systemic infections with hitherto unknown fungi were diagnosed in patients with cancers and chemotherapy-induced neutropenia (61). Pulmonary infections with another fungus, *Pneumocystis*, were diagnosed in patients on immunosuppression following organ transplantation (62, 63). These and other new infections were described as “opportunistic” (61, 64), implying that they occurred preferentially or exclusively in individuals with an overt immunodeficiency. It is difficult to overestimate the importance of these observations. They provided proof of principle that human leukocytes generally, or their individual subsets, are crucial for host defense. They also clearly validated Dubos’ theory, at an unprecedented mechanistic depth and with an unprecedented number of patients, thereby providing unquestionable proof of causality between acquired immunodeficiency and infectious disease. However, these observations were not widely interpreted in that way. Instead, the concept of opportunistic infections ironically isolated these infections, artificially creating a semantic and conceptual barrier between opportunistic infections in patients on chemotherapy or immunosuppression (with overt immunological abnormalities) and “specific” infections (rare or common) in the general population (without such abnormalities). Nevertheless, infections were occasionally referred to as “idiopathic”, in attempts to stress their unexplained occurrence, particularly when opportunistic infections were diagnosed in patients without any detectable immunodeficiency (65, 66).

### From Cortisone to HIV.

Two decades later, in the 1980s, the HIV pandemic provided a tragic example of infectious diseases secondary to an acquired immunodeficiency (*SI Appendix*, Fig. S1). The immunodeficiency in this case consists principally of a decrease in the number of CD4^+^ T cells in the blood following infection with HIV. Multiple opportunistic infections were diagnosed in these patients, including Kaposi sarcoma and environmental (also referred to as non-tuberculous) mycobacterial disease. Both HIV infection and medical immunosuppression are changing circumstances in a person’s life; both changes underlie detectable and predictable leukocytic disorders, and both changes underlie various severe infections. These two unrelated sets of circumstances clearly showed that a newly acquired, environmentally driven, overt immunological deficit could underlie various and often successive or concomitant severe infectious diseases. However, not all the infections in these patients were opportunistic, as the risk of many specific infections, such as herpes simplex virus disease, staphylococcal disease, and tuberculosis, was greatly increased in patients with these overt deficits. These observations, thus, also logically raised the possibility that patients with any common, specific infection despite an absence of immunosuppression or HIV infection might fall ill due to other, covert immunodeficiencies, if also seen as idiopathic. However, this logical inference was rarely made by the scientific and medical community at the time, for reasons alluded to above and discussed in greater depth below. The dichotomy between opportunistic and specific infections continued to persist at the end of the 1980s. Finally, and ironically, acquired immunodeficiencies proved Dubos right only to a certain extent, as their host genetic component was soon deciphered. It was shown that HIV-infected individuals carrying certain HLA alleles could control the virus for prolonged periods of time (67). Furthermore, a connection was established between these acquired immunodeficiencies and host genetic makeup, as rare individuals exposed to HIV remained uninfected, due to homozygosity for a loss-of-function variant of the HIV coreceptor, CCR5 (*SI Appendix*, Fig. S1). These studies raised a possibility with far-reaching consequences: that even acquired immunodeficiencies may paradoxically be primarily inherited immunodeficiencies.

### Mendelian Infections.

Two lines of research led to impressive progress on the human genetic side, which had been neglected by Dubos. The landmark discovery that the sickle cell trait increases resistance to *Plasmodium falciparum* malaria 10-fold at the population level has never been surpassed by candidate gene or genome-wide approaches. The most notable discovery made by these approaches is three to six times higher levels of resistance to hepatitis C virus infection in individuals carrying a specific haplotype encompassing the type III interferon (IFN) locus (68, 69) (*SI Appendix*, Fig. S1). The modest success of this approach probably results from such population-based studies being more suitable for detecting the impact of death from infection on the genomes of human populations than the impact of human genomic variants on the susceptibility of individuals to microbes (70). In other words, population genetic studies have been of greater evolutionary than physiological relevance. This is partly due to their typologist inspiration, which can hardly accommodate the reality that populations, unlike cells and organisms, are not physiological entities governed by genes. By contrast, studies in the field of IEI have provided considerable support for a host theory of infectious diseases. Following the clinical and immunological description of many IEI beginning in the 1950s, their molecular genetic basis began to be dissected from 1985 onward (*SI Appendix*, Fig. S1). These conditions, which were initially referred to as “primary immunodeficiencies”, were typically rare, Mendelian (i.e. monogenic and fully penetrant), and conferred predisposition to multiple infections (*SI Appendix*, Fig. S1). The discovery of their causal genotypes and immunological mechanisms provided molecular genetic proof that infections can be due to genetic immunodeficiencies with complete penetrance. The next major step forward came in 1996, when the molecular basis of a handful of rare Mendelian susceptibilities to single infections was determined in individuals normally resistant to other infections, often from multiplex and/or consanguineous families (*SI Appendix*, Fig. S1). The prototypical example is Mendelian susceptibility to mycobacterial diseases (MSMD), which was shown to result from a wide range of inborn errors of IFN-γ immunity (71). This advance was inspired by elegant forward genetic studies in plants and mice, from 1986 onward, leading to the identification of *Mx* as a key influenza susceptibility locus in mice (*SI Appendix*, Fig. S1), and of *Nramp1* as a key mycobacterial susceptibility locus in mice (*SI Appendix*, Fig. S1), as well as the discovery of the first loci conferring susceptibility to infection in plants (*SI Appendix*, Fig. S1). These lines of research led to the discovery of determinants of common infections, with rare human *MX* variants underlying severe zoonotic influenza in humans (72) and a common human *TYK2* variant underlying up to 1% of TB cases among Europeans (*SI Appendix*, Fig. S1). Paradoxically, the levels of properdin in the blood of patients with HIV infection or other acquired immunodeficiencies were not found to be low, as Dubos had speculated for other changing circumstances, but an X-linked inborn error of properdin identified in 1982 was found to underlie a selective predisposition to recurrent meningococcal disease (*SI Appendix*, Fig. S1). Inherited deficiencies of human lysozyme have not been reported.

### Monogenic Infections.

The next step forward came in 2007, when it was discovered that sporadic infections can be caused by rare monogenic IEI with incomplete penetrance—i.e., non-Mendelian monogenic disorders (12, 13) (*SI Appendix*, Fig. S1). The first example was provided by the study of herpes simplex virus encephalitis (HSE), a life-threatening and sporadic infectious disease, which is rare, despite being the most common viral encephalitis in the Western world and, perhaps, globally. In 5 to 10% of cases, HSE was found to be due to single-gene inborn errors of brain-intrinsic and neuron-intrinsic immunity, with incomplete penetrance (73). Monogenic infections can be rare, as in patients with HSE or even MSMD, which is a misnomer, as only a few genetic etiologies of MSMD are truly Mendelian (71). They can also be common, as with tuberculosis, which is caused by genetic etiologies of MSMD in rare cases. For a given IEI, the penetrance of tuberculosis is much higher than that of MSMD, because *M. tuberculosis* is about 1,000 times more virulent than bacille Calmette-Guérin (BCG) and environmental mycobacteria. Conversely, the proportion of MSMD cases explained by these etiologies is much higher than for tuberculosis. Finally, a fourth genetic step forward was made with studies of the consequences of homozygosity for a *TYK2* variant that is common minor allele frequency (MAF > 1%) in populations of European descent and selectively impairs the IL-23-dependent production of IFN-γ (*SI Appendix*, Fig. S1). This defect has a very low penetrance for MSMD, accounting for a very small proportion of MSMD cases, but a very high penetrance for tuberculosis, accounting for about 1% of cases of tuberculosis in patients of European descent. Importantly, studies of Mendelian and monogenic infections clarified the molecular and cellular mechanism at work in patients with similar infectious diseases due to acquired immunodeficiencies, such as HIV infection (74). For example, we can now suggest that disease due to weakly virulent mycobacteria in individuals with HIV, and perhaps in most patients, is due to impaired IFN-γ immunity.

### Autoimmune Infections.

Remarkably, some rare IEI also led to the discovery of their autoimmune phenocopies, which may be rare or even surprisingly common (*SI Appendix*, Fig. S1). Inborn errors of multiple cytokines are mimicked clinically by autoantibodies (auto-Abs) neutralizing these cytokines. Auto-Abs against IFN-γ (type II IFN) were the first to be shown to underlie severe disease caused by environmental mycobacteria, mimicking inborn errors of IFN-γ (75, 76). Auto-Abs against IL-17A and IL-17F underlie chronic mucocutaneous candidiasis and mimic inborn errors of IL-17A/F (77, 78). Auto-Abs against IL-6 phenocopy inborn errors of IL-6 and underlie staphylococcal disease (79). The commonest and most striking example is auto-Abs neutralizing IFN-α/β (type I IFNs), which are found in 0.3 to 1% of individuals under 65 y of age, with a frequency reaching 4 to 7% in the elderly population (80). They are present before SARS-CoV-2 infection and account for about 15 to 20% of COVID-19 deaths (80–82) (*SI Appendix*, Fig. S1). They also underlie other rare and common viral illnesses, including critical influenza pneumonia and adverse reactions to the yellow fever live attenuated viral vaccine, in a significant proportion of patients (*SI Appendix*, Fig. S1). Their role in the pathogenesis of critical COVID-19 was discovered after a few patients were found to suffer from critical COVID-19 because of IEI of type I IFN immunity, including previously healthy adults with autosomal recessive IFNAR1 or IRF7 deficiency (83), or because of autoimmune polyendocrinopathy syndrome type I (APS-1), a condition underlying the development of multiple auto-Abs, including auto-Abs neutralizing type I IFNs, due to *AIRE* mutations impairing the deletion of autoreactive T cells in the thymus (84, 85). Interestingly, the production of these auto-Abs can itself be driven by APS-1 and other, related IEI impairing AIRE expression in medullary thymic epithelial cells. Overall, in the decades following the publication of Dubos’ 1955 paper, considerable progress was made, making it possible to link various human genetic lesions mechanistically and causally with various severe infectious diseases.

## Conclusion

### An Eagle’s Blind Spot.

The half-century that followed the publication of Dubos’ paper confirmed and massively amplified the results of the half-century that preceded it. It progressively became clear that life-threatening disease during infection can be driven by human genetic determinants, with specific pathogenic mechanisms deciphered at the molecular, cellular, immunological, and whole-organism levels. These host genetic determinants may underlie not only rare infections but also a significant proportion of at least two common infections, pulmonary tuberculosis and COVID-19 (*SI Appendix*, Fig. S1). Their autoimmune phenocopies may underlie an even larger proportion of cases. The body of evidence for a host genetic effect and the corresponding ideas that Dubos neglected, despite the prior publication of many compelling studies of various types in plants and animals, including humans, to which he must have had access in 1955, and even more so in 1976, when he again discussed these matters (60), have grown and flourished. Dubos’ own idea of changing circumstances, although naive at the time, was also validated, both mechanistically and, at an unprecedented scale, with immunosuppression, from the 1960s onward, and HIV infection from the 1980s onward. Dubos focused on changing circumstances, for which few observational data were available, with those that existed mostly relating to intraindividual variability, and virtually no mechanistic insights. Dubos also overlooked 50 y of compelling host genetic studies. He turned out to be half-right, but I think, with the benefit of hindsight, we can conclude in 2023 that he missed the point, and an opportunity, given the data available to him in 1955. There was no solid reason to accord so much importance to changing circumstances, but there were clearly many good reasons to consider the wide range of compelling host genetic studies, which I think should have been the focus, or at least one of the two aspects considered, in any paper expressing second thoughts on the germ theory in 1955.

### Inside Dubos’ Mind.

How can we interpret this misappreciation of his own field, by one of the sharpest and broad-minded microbiologists of the time? Could it be that genetics was, in his mind, orthogonal to ecology, echoing the debate between “nature” and “nurture” (86)? It is possible that Dubos was blinded by his ecological and environmental-to-host, host-extrinsic theory and that this prevented him from considering a seemingly competing genetic and host-intrinsic host theory. It is also possible that Dubos was considering “event” (e.g., striking a match) rather than “constitutive” (e.g., the presence of oxygen in the house) factors as driving causality (in this example, the house catching fire) (87–90). Alternatively, or additionally, Dubos may have hesitated to cross the Rubicon. Indeed, despite his boldness in the title and abstract of the paper, after expressing his second thoughts on the germ theory, he refrained from contesting the germ theory. He remained a believer. I think that this hesitation tells us much about the mindset of microbiologists at the time. Historically, microbiologists have always found it difficult to search for the root cause of infectious diseases, because they operate within a dogma according to which the microbe is causal—and by inference that only the microbe is causal. When studying infectious diseases, they are naturally driven to study the consequences of infection, rather than the causes of disease, which precede infection. The influence of Koch’s radical version of the germ theory, with his postulates that a given pathogen is found in all patients with the corresponding disease and not in any healthy individuals, cannot be overestimated (91). Koch’s postulates bluntly contradict the reality of the infection enigma, as progressively revealed at the turn of the 20th century, due to the inevitable reality of immense interindividual variability during infection. Microbes being living organisms, unlike other environmental challenges, probably also contributed to this microbe-centered view of infectious diseases. In this context, having second thoughts was one thing, and evoking changing circumstances that, through their mysterious mechanism of action and intraindividual nature keep the microbe almost or sufficiently causal in the absence of another proven cause, and equally so across individuals, was another, but looking the facts in the face and accepting the evidence that defective genes that derail immunity in specific individuals are causal for infectious diseases was probably a step too far for René Dubos. He was not ready to conceive or consider this possibility, let alone actually take this intellectual leap. A cause of disease other than the microbe that was both mechanistically and temporally causal and was present before infection and disease, relegating the microbe to the secondary role of an environmental trigger? That was probably unthinkable for this eminent microbiologist and ecologist.

### The Burden of the Germ Theory.

Dubos’ own reluctance to embrace a host theory of infectious diseases tells us much about the general perception of the infection enigma in the microbiology community in 1955 and even today. René Dubos was certainly one of the few in this crowd having second thoughts, and he was arguably one of the most open-minded scholars among them. Even in 2023, most microbiologists remain uncomfortable with a host theory of infectious diseases—or at least with its wide range of consequences. The infection enigma has never been a favorite topic of interest or study for microbiologists—no more than it is for immunologists, for different reasons, to be discussed elsewhere (11, 15, 92). Both communities have been busy tackling other problems, but they clearly display some reluctance to consider this enigma. In the eyes of microbiologists, infectious diseases are infectious. Most microbiologists are prisoners of the germ theory—or more precisely of Koch’s radical version of it, as Pasteur was more prudent. Dubos and microbiologists of all epochs tend to resist the notion that genetic and immunological factors in the host are universal determinants of infectious diseases, as this would revoke, or at least decrease the importance of the germ theory. It is, admittedly, difficult to measure the weights of causal factors, and comparing the respective contributions of the germ and host theories is intellectually challenging (87–90). Second thoughts, however, were and are insufficient. What was needed was a deconstruction of the germ theory—not a demolition, because the germ remains necessary—and the reconstruction, rather than de novo construction, of a host theory, as host theories had already been proposed before the germ theory came to prominence (11) (*SI Appendix*, Fig. S1). With the identification of both host genetic and host immunological determinants of infectious diseases in a growing proportion of cases, for a growing proportion of infections, there is now, at last, an emerging host theory, perhaps heralding a paradigm shift, to which this essay is itself designed to contribute (93–96). In this new paradigm, the microbe is merely an environmental trigger, like phenylalanine in patients with inherited phenylketonuria (97). Who would see phenylalanine as the cause of phenylketonuria? Taking an immunological example, more related to infections, who would see peanut as the cause of peanut allergy (98)? Living or inert, an environmental trigger remains a trigger. In patients who die from infection, the cause of death lies in the host. Dubos was an extraordinary microbiologist and thinker, but he was not clairvoyant or bold enough to envisage or speak about such a revolution. He was not willing to escort the microbe off stage and allow the host to take center stage, or even to share the spotlight with the host.

### The Burden of Immunology.

A corollary is that both microbiologists and immunologists alike have been reluctant to consider death from infection to attest, by definition, to a clinical immunodeficiency at the whole-body level, regardless of the overt or covert nature of the immunodeficiency at the molecular, cellular, and immunological levels. Without the bias of the germ theory, this notion is obvious. Death from hypoxemia is death due to respiratory failure. Death from a coma is death due to brain failure. The mechanisms and manifestations of the failure of vital organs are clearly delineated. This is not the case for immunity to infection. The simple notion that death from infection attests to an immunodeficiency remains poorly understood and accepted, even without considering death from allergy, inflammation, or autoimmunity, which also implies an “immunodeficiency”, albeit less easily defined and detected. Most members of the scientific and medical community, including microbiologists and immunologists, restrict their definition of immunodeficiency to the detection of immunological abnormalities. Bizarrely, there are still countless papers talking about “death from infection in an immunocompetent individual”. This is the equivalent of talking about “death from respiratory failure in a patient with normal pulmonary function” or “death from coma in a patient with normal brain functions”. By reducing host defense and host deficiency to visible features and abnormalities, respectively, of blood leukocytes and their products (narrowly and therefore falsely defining immunity), these studies propagate a confusion between immunological abnormalities, which may be overt or covert (depending on the techniques of detection used, which, of course, evolve with time and have been enriched by the direct detection of causal genotypes), and the immunodeficiency, which can only rigorously be defined by a severe infectious disease (clinical manifestations in whole organisms). The wide persistence of such an intellectual confusion is astounding.

### From Infection to Immunity.

One explanation for this misconception is that, for half a century, host defense was considered to be ensured by antibodies and their complement, and for the next half-century, it was considered to be ensured by antibodies and leukocytes—primarily lymphocytes, at least in the minds of immunologists. Metchnikoff had lost the political battle with Ehrlich at the turn of the 20th century because his phagocytes did not explain the specificity of vaccination, unlike Ehrlich’s antibodies, and did not, therefore, have any impact on the study of the “antibody enigma” until they were found, about a century later, to present antigens to T lymphocytes (99, 100). However, it is becoming increasingly apparent that all or most of the >400 cell types of the human body probably contribute to host defense. Studies of host defense in plants have long pioneered this idea. We are used to the notion that specific organs ensure specific functions, such as thinking for the brain and breathing for the lungs, but there is no organ or system, not even an “immune system” (rarely defined, and when defined, ranging from adaptive lymphocytes only to leukocytes only, occasionally including liver-produced complement), capable of embodying host defense by itself. Host defense is the work of all the cells in the body (101–104). It is difficult to think of a more misleading notion than the immune system when reflecting on immunity. Revisiting immunity and immunology from the angle of host defense therefore inevitably leads to a reconstruction of these ideas. Instead of the traditional immunological approach of trying to explain infections starting from leukocytes, a more physiological approach that has been very fruitful consists of starting from infections and trying to define the cells involved (105). While avoiding the mechanistic problems inherent to the traditional reductionist approach, this new approach is leading to a holistic and mechanistic reconstruction of immunology—and perhaps to another paradigm shift, to which this essay should also contribute (93–96). This approach relies on forward genetics and can be carried out in plants, invertebrates, mice, and humans (105) (*SI Appendix*, Fig. S1). The multicellular nature of host defense is not surprising. In terms of evolution, host defense is ensured by all the cells of the whole body for the good reason that multicellular organisms have been targeted by microbes since their emergence. Protecting the whole body from microbial attacks is the most difficult physiological task in multicellular eukaryotes. Myriad microbial challenges existed before and during the evolution of multicellular organisms, and, unlike other environmental challenges, microbes have since evolved faster than multicellular organisms. It is no coincidence that life expectancy at birth remained at about 20 y from the dawn of humankind until the conquests following the advent of the germ theory. Immunity is the weakest physiological system at both the individual and population levels. Death from infection has a root cause outside the microbe, in the host, and in the host genome in particular. Nevertheless, it is possible to prevent or cure infection without this knowledge. Despite the scientific weaknesses of the germ theory, at least from my point of view, it has proved incontestably strong in medical terms. This is perhaps the main reason for its privileged status, subject to very few challenges.

## Supplementary Material

Appendix 01 (PDF)

## Data Availability

All study data are included in the article and/or *SI Appendix*.
