# Supplementary material for: From second thoughts on the germ theory to a full-blown host theory
Source: Proc Natl Acad Sci U S A. 2023 Jun 12;120(26):e2301186120. doi: 10.1073/pnas.2301186120 (PMC10293828; doi:10.1073/pnas.2301186120)
Supplement: Supplementary file 1 — Appendix 01 (PDF) [file pnas.2301186120.sapp.pdf]

Supplementary Information for

From second thoughts on the germ theory to a full-blown host theory

Jean-Laurent Casanova

This PDF file includes:

Figure S1

References

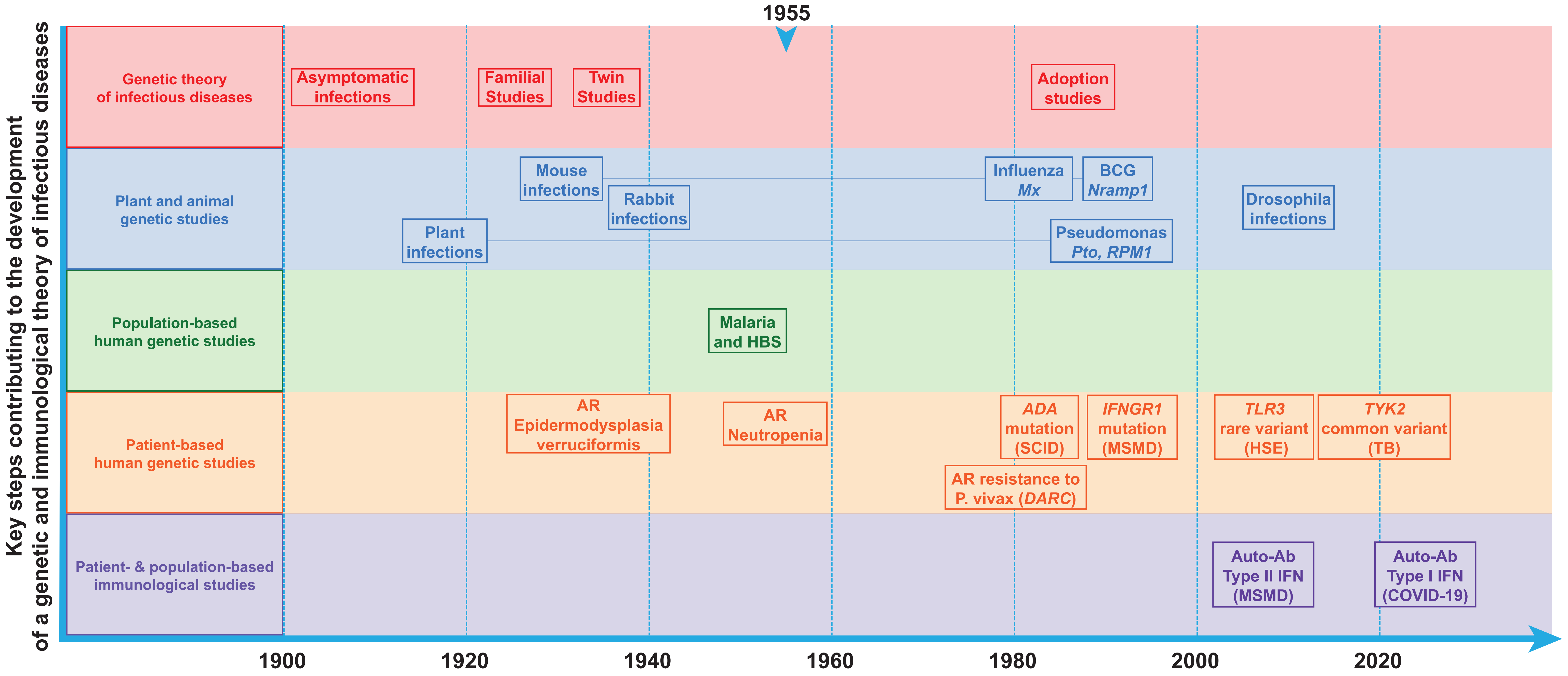
Key steps contributing to the development of a genetic and immunological theory of infectious diseases
1955
Genetic theory of infectious diseases
Asymptomatic infections
Familial Studies
Twin Studies
Adoption studies
Plant and animal genetic studies
Mouse infections
Rabbit infections
Plant infections
Influenza Mx
BCG Nramp1
Pseudomonas Pto, RPM1
Drosophila infections
Population-based human genetic studies
Malaria and HBS
Patient-based human genetic studies
AR Epidermodysplasia verruciformis
AR Neutropenia
ADA mutation (SCID)
IFNGR1 mutation (MSMD)
AR resistance to P. vivax (DARC)
TLR3 rare variant (HSE)
TYK2 common variant (TB)
Patient- & population-based immunological studies
Auto-Ab Type II IFN (MSMD)
Auto-Ab Type I IFN (COVID-19)
1900
1920
1940
1960
1980
2000
2020

Legends for figures

Figure 1 (adapted from (1). Timeline showing some of the key steps that have progressively contributed to the development of a genetic and immunological theory of infectious diseases, prior to and after 1955, grouped into five main parallel fields, from top to bottom: 1) Germ and genetic theories of infectious diseases (red). Following the establishment of the germ theory of disease between 1865 and 1870 (Louis Pasteur) (2), and its radical version with Koch's postulates in 1882 (Robert Koch) (3), it became widely accepted that life-threatening fevers in animals and humans were infectious. Chief among Koch's postulates was the notion that a pathogen must be found in all patients with a given disease and not in healthy individuals. Once established, this postulate made it difficult to appreciate and understand the importance of the evidence of both latent (non-replicating, dormant germs in tissues of asymptomatic individuals; Clemens von Pirquet and followers) (4) and unapparent (replicating germs in tissues or bloodstream of asymptomatic individuals; Charles Nicolle) (5) silent infections. Human geneticists proposed a genetic solution to the problem of asymptomatic infection. Karl Pearson (6) and other population geneticists and biometricians, in parallel with Archibald Garrod (7) and other clinical and biochemical geneticists, proposed that the germline genetic background of the host influences or determines susceptibility or resistance to any given microbe. JBS Haldane proposed that infectious diseases had played a major role in natural selection (8). Various epidemiological and familial approaches were conducted in the first half of the 20th century, supporting this hypothesis. The most remarkable and convincing investigations were probably twin studies comparing monozygotic and dizygotic twins for concordance for a particular infectious phenotype, in particular for tuberculosis (TB) (9). Adoption studies, which compare the cause

of death of patients with that of their biological and foster parents, which are equally powerful, were conducted later (10). Segregation analyses were conducted from the same period onward (11-15).

2) Plant and animal genetic studies (blue). Plant biologists and geneticists also reported early in the 20th century that resistance or susceptibility to infection can be genetically determined (16, 17). From the 1920s onwards, other researchers approached the question of the host genetics of infectious diseases from the angle of animal models, including mice and rabbits. Their results led to a similar conclusion, as some strains were vulnerable to the infections tested, whereas others were not (18, 19). These were experimental, as opposed to natural, infections. The experiments were powerful. By the late 1930s, the stage was set for major progress and a transition to molecular genetics, building on multiple independent lines of thought and investigation. Molecular discoveries began in mice in 1986, with the identification, by molecular complementation, of the first monogenic infection susceptibility gene, *Mx,* mutations of which underlie influenza virus infections (20, 21, Annu Rev Virol 2018), followed by the discoveries, by positional cloning, of the *Bcg* (Nramp1) (22, 23), *Cmv* (Ly49h) (24), and *Lps* (TLR4) (25) loci. In parallel, superb studies unraveled the monogenic basis of various infections in various plant species (26, 27), including, in particular, the role of the *Pto* and *RPM1* genes in resistance to *Pseudomonas* in tomato and *Arabidopsis thaliana*, respectively (28, 29). More recently, the genetic basis of immunity to infection has been studied in diverse species, including the *Caenorhabditis elegans* worm and the *Drosophila melanogaster* fly (30-34). Remarkably, germline deleterious mutations of some genes, such as antimicrobial peptides, seem to impair host defense of Drosophila to specific infections (30-32).

3) Population-based human genetic studies (green). At the population level, the field of human genetics of infectious diseases began with the discovery, by Allison in 1954, that the sickle cell trait (HbS) provides significant protection against severe forms of *P. falciparum* malaria (35). Classical genetic epidemiology studies, particularly segregation analyses followed by genome-wide linkage studies, identified major loci for common infectious conditions, such as schistosomiasis and leprosy (12, 36-38). Very few candidate gene association studies were successful, and only a handful reported odds ratios (ORs) for developing the disease >2 that were replicated in independent populations; among the most remarkable is the association of some HLA class I alleles with AIDS progression, with hazard ratios for protection of about 0.3 (39). Finally, genome-wide association studies (GWAS) met with variable success, depending on the infectious disease, with many loci identified in leprosy (40, 41) with ORs below 2, and, more recently, malaria (42), with ORs for protection above 0.5, except for the *HBB* locus. The most remarkable achievement of GWAS in infectious diseases was probably the identification, towards 2010, of *IL28B* variants (*IL28B* encodes the type III IFN-λ3) strongly associated with the clearance of hepatitis C virus (43) (OR for clearance ~6). More recently, GWAS in COVID-19 have identified several common variants associated with severe pneumonia, the most significant being located on chromosome 3 with an OR ~2 (44-46).

4) Patient-based human genetic studies (orange). In the early 1950s, pediatricians and clinical geneticists described the first inborn errors of immunity (IEIs) — then referred to as primary immunodeficiencies (PIDs) — as rare, Mendelian, early-onset conditions underlying both multiple, recurrent, and opportunistic infections, and overt, or at least detectable, immunological abnormalities. The blueprint for conventional IEI is widely agreed to be the description of Bruton's X-linked recessive (XR) agammaglobulinemia in 1952 (47). Severe

congenital neutropenia had, however, been described earlier, in 1950, in children with severe staphylococcal and other bacterial infections and congenital neutropenia, two phenotypes that co-segregated as an autosomal recessive (AR) trait (48, 49). The first IEI conferring predisposition to a single infectious agent was, however, reported even earlier, in 1946, when Wilhelm Lutz described epidermodysplasia verruciformis (EV) as an AR predisposition to skin-tropic viruses (50) identified in 1978 by Gérard Orth to be weakly virulent human papillomaviruses (HPV) (51). Immunologists did not consider EV to be an IEI, because of the lack of a detectable leukocyte abnormality, until the discovery of EV-causing genes from 2002 onward led to the gradual recognition that keratinocytes contribute to host defense (52, 53). Defects of the membrane attack complex of complement (underlying infections with *Neisseria*), and X-linked lymphoproliferation (XLP, Epstein-Barr virus) were described later, from different angles, as XLP, like EV, was described as a Mendelian and unexplained (idiopathic) infection (54, 55), whereas complement defects were found serendipitously in sporadic cases of *Neisseria* disease (56, 57). X-linked recessive deficiency of properdin and the alternative pathway of complement activation was first described in 1982 (58). Mendelian resistance to infectious agents was first detected in the 1970s, with the Duffy antigen receptor for chemokines (DARC) and *P. vivax* malaria (59), the genetic basis of which was elucidated in 1995 (60). It was followed, in the mid-1990s, by the discovery of the link between C-C chemokine receptor 5 (CCR5) variants and resistance to human immunodeficiency virus-1 (HIV-1) (61, 62, 63, Science). The first mutated gene underlying a conventional IEI, *ADA*, was reported in patients with severe combined immunodeficiency in 1985 (64). The first mutated gene underlying an isolated infection, *IFNGR1*, was identified in patients with MSMD in 1996 (65, 66). Since 2007, some rare and sporadic infectious diseases (e.g. HSE) have been shown to be caused by rare monogenic defects, with incomplete

penetrance (67, 68). Since 2001, rare monogenic defects have also been shown to underlie some common infectious diseases in rare patients (e.g. TB in 2001 (69) and COVID-19 in 2020 (70)). Since 2018, monogenic causes of infection have been found in a greater proportion of patients with TB (due to homozygosity for a common *TYK2* allele) (71-73) or COVID-19 (due to hemizygosity for various rare *TLR7* alleles) (67, 74).

5) Patient- and population-based immunological studies (magenta). Acquired immunodeficiency and ensuing infections, due to chemotherapy for malignancies or immunosuppression for organ transplant or autoimmune conditions became widely documented from the 1960s onward (35, 75, 76, Immunopharmacology 2020). Another step was taken from the 1980s onward, with the human immunodeficiency virus pandemic (77). Auto-Abs against four cytokines (type I and II IFNs, IL-6, and IL-17A/F) have been shown to underlie infectious phenocopies of the corresponding inborn errors of cytokines or their response pathways (78, 79). The best characterized are probably auto-Abs neutralizing type II IFN, which underlie a phenocopy of MSMD and, more rarely, TB (79, 80). Auto-Abs neutralizing type I IFNs, first described in the early 1980s, were long thought to be clinically silent, except for a 77-year-old woman with disseminated zoster studied by Ion Gresser (81, 82). They were found in almost all patients with autoimmune polyendocrine syndrome type 1 (APS-1) (83, 84). Since 2020, they have been shown to be strong determinants of at least 15% of cases of life-threatening COVID-19 pneumonia (85-87) and a third of adverse reactions to the live-attenuated yellow fever vaccine (88). More recently, they have also been shown to account for ~20% of hypoxemic breakthrough COVID-19 cases in fully vaccinated subjects (89), and ~5% of cases of severe influenza pneumonia in patients younger than 70 years (90).

References

1. J. L. Casanova, L. Abel, From rare disorders of immunity to common determinants of infection: Following the mechanistic thread. *Cell* **185**, 3086-3103 (2022).
2. L. Pasteur, *Oeuvres complètes de Louis Pasteur, réunies par Pasteur Vallery-Radot* (Masson et Cie, Paris, 1922-1939).
3. R. Koch, Die Aetiologie der Tuberkulose. *Berl Klin Wochenschr* **19**, 221-230 (1882).
4. A. Alcais, M. Mira, J. L. Casanova, E. Schurr, L. Abel, Genetic dissection of immunity in leprosy. *Curr Opin Immunol* **17**, 44-48 (2005).
5. C. Nicolle, Les infections inapparentes. *Scientia*, 181-271 (1933).
6. K. Pearson (1912) Tuberculosis, heredity and environment. (Dulau and Co., LTD., The Galton laboratory for national eugenics), p 46.
7. A. E. Garrod, *The Inborn Factors in Disease* (Clarendon Press, Oxford, 1931).
8. J. B. Haldane, Suggestions as to quantitative measurement of rates of evolution. *Ric Sci* **3**, 51-56 (1949).
9. R. Puffer, *Familial susceptibility to tuberculosis; Its importance as a public health problem*, Harvard University monographs in medicine and public health (Harvard University Press, Cambridge, MA, 1944), vol. 5, pp. 106.
10. T. I. Sorensen, G. G. Nielsen, P. K. Andersen, T. W. Teasdale, Genetic and environmental influences on premature death in adult adoptees. *N Engl J Med* **318**, 727-732 (1988).
11. L. Abel, M. Cot, L. Mulder, P. Carnevale, J. Feingold, Segregation analysis detects a major gene controlling blood infection levels in human malaria. *Am J Hum Genet* **50**, 1308-1317 (1992).
12. L. Abel, F. Demenais, Detection of major genes for susceptibility to leprosy and its subtypes in a Caribbean island: Desirade island. *Am J Hum Genet* **42**, 256-266 (1988).
13. L. Abel, F. Demenais, A. Prata, A. E. Souza, A. Dessein, Evidence for the segregation of a major gene in human susceptibility/resistance to infection by Schistosoma mansoni. *Am J Hum Genet* **48**, 959-970 (1991).
14. S. Plancoulaine *et al.*, Detection of a major gene predisposing to human T lymphotropic virus type I infection in children among an endemic population of African origin. *J Infect Dis* **182**, 405-412 (2000).
15. D. K. Wagener *et al.*, Segregation analysis of leprosy in families of northern Thailand. *Genet Epidemiol* **5**, 95-105 (1988).
16. R. H. Biffen, Mendel's laws of inheritance and wheat breeding. *J Agricult Science* **1**, 4-48 (1905).
17. H. H. Flor, Inheritance of pathogenicity in a cross between physiologic races 22 and 24 of Melampsora lini. *Phytopathology* **32**, 5 (1942).
18. M. Lurie, Heredity, constitution and tuberculosis: an experimental study. *Am Rev Tuberc* **44(suppl)**, 1-125 (1941).
19. L. T. Webster, Heredity in Infectious Disease. *J Heredity* **30**, 365-370 (1939).
20. P. Staeheli, O. Haller, W. Boll, J. Lindenmann, C. Weissmann, Mx protein: constitutive expression in 3T3 cells transformed with cloned Mx cDNA confers selective resistance to influenza virus. *Cell* **44**, 147-158 (1986).
21. O. Haller, H. Arnheiter, J. Pavlovic, P. Staeheli, The Discovery of the Antiviral Resistance Gene Mx: A Story of Great Ideas, Great Failures, and Some Success. *Annu Rev Virol* **5**, 33-51 (2018).

22. E. Skamene *et al.*, Genetic regulation of resistance to intracellular pathogens. *Nature* **297**, 506-509 (1982).
23. S. M. Vidal, D. Malo, K. Vogan, E. Skamene, P. Gros, Natural resistance to infection with intracellular parasites: isolation of a candidate for Bcg. *Cell* **73**, 469-485 (1993).
24. M. G. Brown *et al.*, Vital involvement of a natural killer cell activation receptor in resistance to viral infection. *Science* **292**, 934-937 (2001).
25. A. Poltorak *et al.*, Defective LPS signaling in C3H/HeJ and C57BL/10ScCr mice: mutations in Tlr4 gene. *Science* **282**, 2085-2088 (1998).
26. J. L. Dangl, J. D. Jones, Plant pathogens and integrated defence responses to infection. *Nature* **411**, 826-833 (2001).
27. J. D. Jones, R. E. Vance, J. L. Dangl, Intracellular innate immune surveillance devices in plants and animals. *Science* **354**, eaaf6395 (2016).
28. M. R. Grant *et al.*, Structure of the Arabidopsis RPM1 gene enabling dual specificity disease resistance. *Science* **269**, 843-846 (1995).
29. G. B. Martin *et al.*, Map-based cloning of a protein kinase gene conferring disease resistance in tomato. *Science* **262**, 1432-1436 (1993).
30. M. A. Hanson, B. Lemaitre, New insights on Drosophila antimicrobial peptide function in host defense and beyond. *Curr Opin Immunol* **62**, 22-30 (2020).
31. B. Lemaitre, J. Hoffmann, The host defense of Drosophila melanogaster. *Annu Rev Immunol* **25**, 697-743 (2007).
32. B. Lemaitre, E. Nicolas, L. Michaut, J. M. Reichhart, J. A. Hoffmann, The dorsoventral regulatory gene cassette spatzle/Toll/cactus controls the potent antifungal response in Drosophila adults. *Cell* **86**, 973-983 (1996).
33. E. Pradel, J. J. Ewbank, Genetic models in pathogenesis. *Annu Rev Genet* **38**, 347-363 (2004).
34. N. Pujol, J. J. Ewbank, C. elegans: out on an evolutionary limb. *Immunogenetics* **74**, 63-73 (2022).
35. A. C. Allison, Protection afforded by sickle-cell trait against subtertian malareal infection. *Br Med J* **1**, 290-294 (1954).
36. A. Alcais *et al.*, Stepwise replication identifies a low-producing lymphotoxin-alpha allele as a major risk factor for early-onset leprosy. *Nat Genet* **39**, 517-522 (2007).
37. S. Marquet *et al.*, Genetic localization of a locus controlling the intensity of infection by *Schistosoma mansoni* on chromosome 5q31-q33. *Nat Genet* **14**, 181-184 (1996).
38. M. T. Mira *et al.*, Susceptibility to leprosy is associated with PARK2 and PACRG. *Nature* **427**, 636-640 (2004).
39. R. A. Kaslow *et al.*, Influence of combinations of human major histocompatibility complex genes on the course of HIV-1 infection. *Nat Med* **2**, 405-411 (1996).
40. F. Zhang *et al.*, Identification of two new loci at IL23R and RAB32 that influence susceptibility to leprosy. *Nat Genet* **43**, 1247-1251 (2011).
41. F. R. Zhang *et al.*, Genomewide association study of leprosy. *N Engl J Med* **361**, 2609-2618 (2009).
42. N. Malaria Genomic Epidemiology, Insights into malaria susceptibility using genome-wide data on 17,000 individuals from Africa, Asia and Oceania. *Nat Commun* **10**, 5732 (2019).
43. D. Ge *et al.*, Genetic variation in IL28B predicts hepatitis C treatment-induced viral clearance. *Nature* **461**, 399-401 (2009).

44. C.-H. G. Initiative, Mapping the human genetic architecture of COVID-19. *Nature* **600**, 472-477 (2021).
45. E. Pairo-Castineira *et al.*, Genetic mechanisms of critical illness in COVID-19. *Nature* **591**, 92-98 (2021).
46. A. Kousathanas *et al.*, Whole-genome sequencing reveals host factors underlying critical COVID-19. *Nature* **607**, 97-103 (2022).
47. O. C. Bruton, Agammaglobulinemia. *Pediatrics* **9**, 722-728 (1952).
48. R. Kostmann, Hereditär reticulos - en ny systemsjukdom. *Svenska Läkartdin* **47**, 2861 (1950).
49. R. Kostmann, Infantile genetic agranulocytosis; agranulocytosis infantilis hereditaria. *Acta Paediatr Suppl (Upps)* **45**, 1-78 (1956).
50. W. Lutz, A propos de l'épidermodysplasie verruciforme. *Dermatologica* **92**, 30-43 (1946).
51. G. Orth *et al.*, Characterization of two types of human papillomaviruses in lesions of epidermodysplasia verruciformis. *Proc Natl Acad Sci U S A* **75**, 1537-1541 (1978).
52. N. Ramoz *et al.*, Mutations in two adjacent novel genes are associated with epidermodysplasia verruciformis. *Nat Genet* **32**, 579-581 (2002).
53. S. J. de Jong *et al.*, The human CIB1-EVER1-EVER2 complex governs keratinocyte-intrinsic immunity to beta-papillomaviruses. *J Exp Med* **215**, 2289-2310 (2018).
54. D. T. Purtilo, C. Cassel, J. P. Yang, Letter: Fatal infectious mononucleosis in familial lymphohistiocytosis. *N Engl J Med* **291**, 736 (1974).
55. D. T. Purtilo *et al.*, Variable phenotypic expression of an X-linked recessive lymphoproliferative syndrome. *N Engl J Med* **297**, 1077-1080 (1977).
56. D. Lim *et al.*, Absence of the sixth component of complement in a patient with repeated episodes of meningococcal meningitis. *J Pediatr* **89**, 42-47 (1976).
57. B. H. Petersen, J. A. Graham, G. F. Brooks, Human deficiency of the eighth component of complement. The requirement of C8 for serum Neisseria gonorrhoeae bactericidal activity. *J Clin Invest* **57**, 283-290 (1976).
58. A. G. Sjoholm, J. H. Braconier, C. Soderstrom, Properdin deficiency in a family with fulminant meningococcal infections. *Clin Exp Immunol* **50**, 291-297 (1982).
59. L. H. Miller, S. J. Mason, D. F. Clyde, M. H. McGinniss, The resistance factor to Plasmodium vivax in blacks. The Duffy-blood-group genotype, FyFy. *N Engl J Med* **295**, 302-304 (1976).
60. C. Tournamille, Y. Colin, J. P. Cartron, C. Le Van Kim, Disruption of a GATA motif in the Duffy gene promoter abolishes erythroid gene expression in Duffy-negative individuals. *Nat Genet* **10**, 224-228 (1995).
61. R. Liu *et al.*, Homozygous defect in HIV-1 coreceptor accounts for resistance of some multiply-exposed individuals to HIV-1 infection. *Cell* **86**, 367-377 (1996).
62. M. Samson *et al.*, Resistance to HIV-1 infection in caucasian individuals bearing mutant alleles of the CCR-5 chemokine receptor gene. *Nature* **382**, 722-725 (1996).
63. M. Dean *et al.*, Genetic restriction of HIV-1 infection and progression to AIDS by a deletion allele of the CKR5 structural gene. Hemophilia Growth and Development Study, Multicenter AIDS Cohort Study, Multicenter Hemophilia Cohort Study, San Francisco City Cohort, ALIVE Study. *Science* **273**, 1856-1862 (1996).
64. D. T. Bonthron, A. F. Markham, D. Ginsburg, S. H. Orkin, Identification of a point mutation in the adenosine deaminase gene responsible for immunodeficiency. *J Clin Invest* **76**, 894-897 (1985).

65. E. Jouanguy *et al.*, Interferon-gamma-receptor deficiency in an infant with fatal bacille Calmette-Guerin infection. *N Engl J Med* **335**, 1956-1961 (1996).
66. M. J. Newport *et al.*, A mutation in the interferon-gamma-receptor gene and susceptibility to mycobacterial infection. *N Engl J Med* **335**, 1941-1949 (1996).
67. J. L. Casanova, L. Abel, Lethal Infectious Diseases as Inborn Errors of Immunity: Toward a Synthesis of the Germ and Genetic Theories. *Annu Rev Pathol* **16**, 23-50 (2021).
68. S. Y. Zhang *et al.*, TLR3 deficiency in patients with herpes simplex encephalitis. *Science* **317**, 1522-1527 (2007).
69. F. Altare *et al.*, Interleukin-12 receptor beta1 deficiency in a patient with abdominal tuberculosis. *J Infect Dis* **184**, 231-236 (2001).
70. Q. Zhang *et al.*, Inborn errors of type I IFN immunity in patients with life-threatening COVID-19. *Science* **370**, eabd4570 (2020).
71. S. Boisson-Dupuis *et al.*, Tuberculosis and impaired IL-23-dependent IFN-gamma immunity in humans homozygous for a common TYK2 missense variant. *Sci Immunol* **3**, eaau8714 (2018).
72. G. Kerner *et al.*, Homozygosity for TYK2 P1104A underlies tuberculosis in about 1% of patients in a cohort of European ancestry. *Proc Natl Acad Sci U S A* **116**, 10430-10434 (2019).
73. G. Kerner *et al.*, Human ancient DNA analyses reveal the high burden of tuberculosis in Europeans over the last 2,000 years. *Am J Hum Genet* **108**, 517-524 (2021).
74. T. Asano *et al.*, X-linked recessive TLR7 deficiency in ~1% of men under 60 years old with life-threatening COVID-19. *Sci Immunol* **6**, eabl4348 (2021).
75. V. T. DeVita, Jr., E. Chu, A history of cancer chemotherapy. *Cancer Res* **68**, 8643-8653 (2008).
76. A. C. Allison, Immunosuppressive drugs: the first 50 years and a glance forward. *Immunopharmacology* **47**, 63-83 (2000).
77. S. M. Schnittman, A. S. Fauci, Human immunodeficiency virus and acquired immunodeficiency syndrome: an update. *Adv Intern Med* **39**, 305-355 (1994).
78. C. L. Ku, C. Y. Chi, H. von Bernuth, R. Doffinger, Autoantibodies against cytokines: phenocopies of primary immunodeficiencies? *Hum Genet* **139**, 783-794 (2020).
79. A. Puel, P. Bastard, J. Bustamante, J. L. Casanova, Human autoantibodies underlying infectious diseases. *J Exp Med* **219**, e20211387 (2022).
80. H. P. Shih, J. Y. Ding, C. F. Yeh, C. Y. Chi, C. L. Ku, Anti-interferon-gamma autoantibody-associated immunodeficiency. *Curr Opin Immunol* **72**, 206-214 (2021).
81. K. E. Mogensen, P. Daubas, I. Gresser, D. Sereni, B. Varet, Patient with circulating antibodies to alpha-interferon. *Lancet* **2**, 1227-1228 (1981).
82. B. Pozzetto, K. E. Mogensen, M. G. Tovey, I. Gresser, Characteristics of autoantibodies to human interferon in a patient with varicella-zoster disease. *J Infect Dis* **150**, 707-713 (1984).
83. E. S. Husebye, M. S. Anderson, O. Kampe, Autoimmune Polyendocrine Syndromes. *N Engl J Med* **378**, 2543-2544 (2018).
84. A. Meager *et al.*, Anti-interferon autoantibodies in autoimmune polyendocrinopathy syndrome type 1. *PLoS Med* **3**, e289 (2006).
85. P. Bastard *et al.*, Autoantibodies neutralizing type I IFNs are present in ~4% of uninfected individuals over 70 years old and account for ~20% of COVID-19 deaths. *Sci Immunol* **6**, eabl4340 (2021).

86. P. Bastard *et al.*, Autoantibodies against type I IFNs in patients with life-threatening COVID-19. *Science* **370**, eabd4585 (2020).
87. Q. Zhang, P. Bastard, C. H. G. Effort, A. Cobat, J. L. Casanova, Human genetic and immunological determinants of critical COVID-19 pneumonia. *Nature* **603**, 587-598 (2022).
88. P. Bastard *et al.*, Auto-antibodies to type I IFNs can underlie adverse reactions to yellow fever live attenuated vaccine. *J Exp Med* **218**, e20202486 (2021).
89. J. Rosain *et al.*, Mendelian susceptibility to mycobacterial disease: 2014-2018 update. *Immunol Cell Biol* **97**, 360-367 (2019).
90. Q. Zhang *et al.*, Recessive inborn errors of type I IFN immunity in children with COVID-19 pneumonia. *J Exp Med* **219**, e20220131 (2022).
